# Disparities in chronic ischemic heart disease-related mortality across sex, race, and urbanization status in the United States, 1999–2019

**DOI:** 10.1371/journal.pgph.0004705

**Published:** 2025-09-11

**Authors:** Patrick A. Kwaah, Samuel A. Mensah, Abraham Carboo, Grace Appah, Hamza A. Rashid, Stephen N. Djanie, Ama O. Kwakye, Emmanuel A. Agyemang, Joseph S. Kekrebesi, Webster Donaldy, Patrick Nti, Christopher B. Sarpong

**Affiliations:** 1 Department of Internal Medicine, Yale School of Medicine, Waterbury, Connecticut, United States of America; 2 Department of Internal Medicine, West Virginia University, Morgantown, West Virginia, United States of America; 3 Department of Internal Medicine, Korlebu Teaching Hospital, Accra, Ghana; 4 Department of Internal Medicine, University of Cape Coast, Cape Coast, Ghana; 5 Department of Internal Medicine, Newark Beth Israel Medical Center, Newark, New Jersey, United States of America; 6 Division of Cardiology, Emory University School of Medicine, Atlanta, Georgia, United States of America; 7 Department of Internal Medicine, Harlem Hospital Center, Columbia University, New York, New York, United States of America; 8 Department of Internal Medicine, 37 Military Hospital, Accra, Ghana; Universiti Malaya, MALAYSIA

## Abstract

Chronic ischemic heart disease (CIHD) is one of the leading causes of significant morbidity and mortality in the United States. While previous studies have reported an overall decline in ischemic heart disease mortality, contemporary trends in CIHD-related mortality across sex, race, and urbanization status remain inadequately explored. We examined CIHD-related mortality trends in the U.S. from 1999 to 2019.We analyzed mortality data from the CDC WONDER database using CIHD ICD-10 codes. Age-adjusted mortality rates (AAMR) were calculated per 100,000 individuals. Trends were analyzed using Joinpoint regression to determine annual percentage change (APC) and average annual percentage change (AAPC) with 95% confidence intervals. Over two decades, 5,729,619 CIHD-related deaths were recorded. AAMR declined from 185.6 (95% CI: 184.9–186.2) per 100,000 in 1999 to 94.9 (95% CI: 94.5–95.3) per 100,000 in 2019. There were, however, disparities among demographic groups. Males had consistently higher mortality than females (overall AAMR: 167.2 vs. 96.0 per 100,000), and among racial groups, non-Hispanic Black individuals had the highest AAMR (148.3 per 100,000. Initially, urban areas had higher mortality than rural areas, but by 2019, their AAMRs converged (urban: 94.7 [95% CI: 94.3–95.1]; rural: 96.1 [95% CI: 95.1–97.0] per 100,000). CIHD mortality has declined across all demographics over the last two decades; however, disparities persist, particularly among males and non-Hispanic Black individuals. While rural and urban populations had differing mortality rates initially, they showed similar AAMR by the end of the study period. Focused public health interventions are crucial to addressing these inequities.

## Introduction

Ischemic heart disease (IHD) is a heterogeneous group of cardiac diseases characterized by reduced perfusion and oxygen supply to cardiac myocytes [1]. Chronic ischemic heart disease (CIHD) is a subset of IHD that results from the gradual accumulation of plaque in the coronary arteries, leading to narrowing and subsequent ischemia of the myocardium. This entity is distinct from acute ischemic heart disease, which is caused by plaque rupture, thrombotic occlusion, or coronary vessel constriction [2]. Established risk factors for IHD include non-modifiable factors such as advancing age, male sex, ethnicity, and family history, as well as modifiable factors like hypertension, hyperlipidemia, diabetes, smoking, and obesity [1,3]. Globally, the prevalence of IHD was estimated at 197 million in 2019, with 9.14 million deaths and 182 million disability-adjusted life years (DALYs). In the United States (U.S.), IHD is the leading cause of mortality, accounting for about 610,000 deaths annually, with over 200 billion dollars in overall healthcare services cost [4]. Over the past two decades, significant advancements have been made in the diagnosis and treatment of ischemic heart disease (IHD). These include the development of innovative diagnostic tools, such as quantitative flow ratio and novel biomarkers, to improve the prognostication of atherosclerosis [5]. Despite these advancements and an overall reduction in IHD and acute myocardial infarction (AMI) mortality there is limited data regarding the mortality trends of chronic ischemic heart disease (CIHD) [6,7]. A large European database study examining IHD mortality trends observed that the decline in IHD related mortality predominantly reflects reductions in acute IHD as opposed to CIHD [8].To address this gap, we aimed to investigate CIHD-related mortality trends in the U.S., focusing on disparities across demographic and geographic populations. Understanding these trends is critical, as this may highlight healthcare-related barriers impacting the prevention and treatment of this high-mortality disease. The objectives of this study are: [1] to assess trends in CIHD-related mortality across sex and race/ethnicity, and [2] to examine rural-urban differences in CIHD-related mortality.

## Methodology

We analyzed mortality data from the Centers for Disease Control and Prevention’s Wide-Ranging Online Data for Epidemiologic Research (CDC WONDER) database from 1999 to 2019. This mortality dataset includes information from death certificates across all 50 states and the District of Columbia, capturing demographic details as well as underlying and multiple causes of death. We identified individuals who died from CIHD using codes I25.0 - I25.6 and I25.8, and I25.9 [9,10]. Counties were classified into rural and urban categories according to the 2013 National Center for Health Statistics Urban-Rural Classification Scheme. Rural areas (population <50,000) included micropolitan and non-core areas, while urban areas (population ≥ 50,000) were categorized as large central metropolitan, large fringe metropolitan, medium metropolitan, and small metropolitan areas [11]. Demographic variables included gender, age, race, ethnicity, and census region (Northeast, South, Midwest, and West). Ethnicity was categorized as non-Hispanic White (NHW), non-Hispanic Black (NHB), Hispanic, and non-Hispanic others (NHO). Due to limited deaths at certain time points, Alaskan Native, American Indian, and Asian or Pacific Islander were combined into the non-Hispanic other race/ethnicity category to prevent rate suppression [12]. Annual CIHD-mortality rates were defined as the number of deaths per 100,000 population by rural-urban classification and demographic factors. Age-Adjusted Mortality Rates (AAMR) per 100,000 individuals were calculated by adjusting age-specific mortality rates to the 2000 US standard population. The annual percent change (APC) and average annual percentage changes (AAPC) in AAMR, with 95% confidence intervals (CI), were calculated using the Joinpoint Regression Program (Version 5.2.0, National Cancer Institute). Joinpoint regression modeling assesses temporal trends in mortality rates by identifying points in time where there is a statistical change in the linear slope of the trend. It does this by fitting log-linear models to the age-adjusted rates, and the slope of each segment is used to calculate APC using the formula APC = (e^β − 1) × 100, where β is the estimated slope from the regression model. The AAPC is derived as a weighted average of the APCs across the full-time span, with weights based on the length of each segment. Statistical significance was assessed using a Monte Carlo permutation method, and two-sided p-values <0.05 were considered significant [13,14]. This study was exempt from Institutional Review Board approval by the CDC as it utilized de-identified, publicly accessible data.

## Results

Between 1999 and 2019, a total of 5729619 CIHD-related deaths occurred among adults 25 years and above in the United States. Overall, CIHD-related mortality showed a substantial decrease throughout the study period, with AAMR of 185.6 (95% CI: 184.9 to 186.2) in 1999 and 94.9 (95% CI: 94.5 to 95.3) in 2019. Most of the deaths occurred in males (53.7%), urban settings (83.5%), and among the NHW population (81.7%) (Table 1,2).

**Table 1:** Characteristics and trends in the age-adjusted mortality rate of individuals ≥25 years who had CIHD as the underlying cause of death in the United States, 1999 – 2019.

	Deaths:	Proportion of individuals %	AAMR for CIHD per 100, 0000 population	Change in AAMR Trends from 1999-2019 (%)	CIHD AAMR AAPC (%) (95% Confidence interval)
Overall	5729619	100	126.8	185.6 to 94.9 (-48.9)	-3.27 (-3.34, -3.20)
**Gender**					
Female	2651387	46.3	96	147.6 to 66.0 (-55.3)	-3.91 (-3.99, -3.83)
Male	3078232	53.7	167.2	237.2 to 131.6 (-44.5)	-2.88 (-2.95, -2.80)
**Race**					
Hispanic or Latino	300323	5.2	97.8	161.6 to 73.2 (-54.7)	-3.94 (-4.14, -3.67)
Non-Hispanic Black	591693	10.3	148.3	217.2 to 110.1 (-49.3)	-3.31 (-3.39, -3.21)
Non-Hispanic Other	137507	2.4	75.9	120.8 to 58.8 (-51.3)	-3.39 (-3.63, -3.20)
Non-Hispanic White	4678866	81.7	128.5	184.2 to 97.3 (47.0)	-3.12 (-3.18, -3.04)
**Community Setting**					
Rural	946890	16.5	118.8	160.0 to 96.1 (-39.9)	-2.49 (-2.59, -2.37)
Urban	4782729	83.5	128.6	191.7 to 94.7 (-50.6)	-3.44 (-3.51, -3.36)

AAMR, age adjusted mortality rate; CIHD, chronic ischemic heart disease.

**Table 2:** Annual Mortality Rates of CIHD Among Individuals Aged ≥25 Years in the United States, 1999–2019.

Year	Deaths	Population	Crude Rate	AAMR	AAMR Lower 95% CI	AAMR Upper 95% CI
1999	326342	180408769	180.9	185.6	184.9	186.2
2000	318415	181984640	175	178.6	178	179.2
2001	313545	184305128	170.1	172.9	172.3	173.5
2002	310966	186208028	167	168.9	168.3	169.5
2003	305889	188090429	162.6	163.3	162.7	163.9
2004	291709	190205384	153.4	153.3	152.8	153.9
2005	290774	192551384	151	149.6	149	150.1
2006	279711	195019359	143.4	140.7	140.2	141.3
2007	268976	197403777	136.3	132.4	131.9	132.9
2008	266812	199795090	133.5	128.5	128	129
2009	256569	202107016	126.9	121	120.5	121.4
2010	253089	203891983	124.1	117.2	116.7	117.7
2011	251154	206592936	121.6	112.9	112.5	113.4
2012	249468	208826037	119.5	109.4	109	109.9
2013	249196	211085314	118.1	106.8	106.4	107.2
2014	246271	213809280	115.2	103.2	102.8	103.6
2015	248367	216553817	114.7	101.8	101.4	102.2
2016	247332	218641417	113.1	99.2	98.8	99.6
2017	250776	221447331	113.2	98.4	98	98.8
2018	252399	223311190	113	96.9	96.5	97.3
2019	251859	224981167	111.9	94.9	94.5	95.3
**TOTAL**	**5729619**	**4247219476**	**134.9**	**126.8**	**126.7**	**126.9**

CIHD, chronic ischemic heart disease; AAMR, age adjusted mortality rate; CI, confidence interval.

### Cihd- related mortality stratified by gender

**Fig 1:**
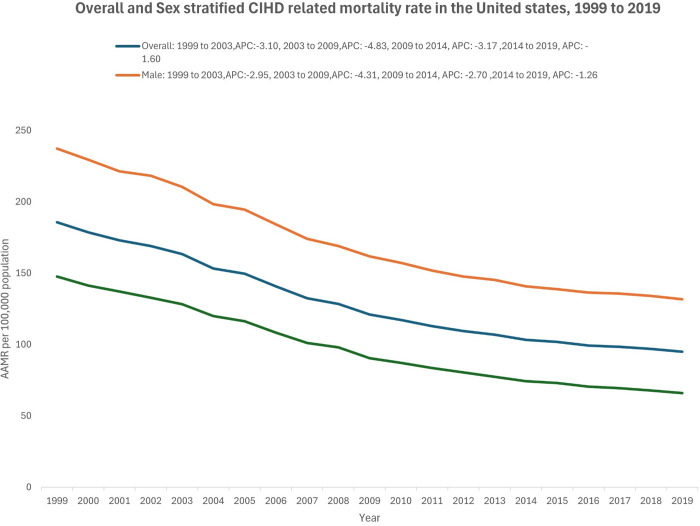
Overall and sex stratified CIHD related age adjusted mortality rate in the United States, 1999 to 2019.

There was a decline in the mortality rate for both males and females over the two-decade period. However, males consistently had higher age-adjusted mortality rates (AAMRs) than females (Fig 1). Overall, males had an AAMR of 167.2 (95% CI: 167.0 to 167.4) compared to females, whose AAMR was 96.0 (95% CI: 95.9 to 96.2). For males, the AAMR started at 237.2 (95% CI: 236.0 to 238.4) in 1999 and decreased to 131.6 (95% CI: 130.9 to 132.3) by 2019, with an overall AAPC of -2.88 (95% CI: -2.95 to -2.80, p < 0.01) (Table 1,3). There was an initial decline from 1999 to 2003 APC: -2.95, p < 0.01), followed by a steeper decline from 2003 to 2009 (APC: -4.31, p < 0.01), a further decrease from 2009 to 2014 (APC: -2.70, p < 0.01), and a slower decrease from 2014 to 2019 (APC: -1.26, p < 0.01)(Fig 1, Table 3). Imitating this trend, the AAMR for females declined from 147.6 (95% CI: 146.9 – 148.3) in 1999 to 66.0 (95% CI: 65.6 – 66.4) in 2019 with an AAPC of -3.91 (95% CI:-3.99, -3.83, p: < 0.01) (Table 1). The APC showed a decline from 1999 to 2003 (APC: -3.31, p < 0.01), followed by the steepest decrease from 2003 to 2009 (APC: -5.37, p < 0.01), a further decrease from 2009 to 2014 (APC: -4.00, p < 0.01), and a smaller decline from 2014 to 2019 (APC: -2.29, p < 0.01). (Fig 1, Table 3)

**Table 3:** Annual percent change of CIHD AAMR in the United States, 1999 to 2019.

Year Interval	APC (95% CI)
**Overall**	
1999-2003	-3.10* (-3.54, -2.46)
2003-2009	-4.84 (-5.35, -4.58)
2009-2014	-3.17* (-3.67, -2.71)
2014-2019	-1.60 * (-1.92, -1.14)
**Male**	
1999-2003	-2.95*(-3.37, -2.24)
2003-2009	-4.31* (-4.88, -4.05)
2009-2014	-2.70* (-3.31, -2.19)
2014-2019	-1.26* (-1.60, -0.68)
**Female**	
1999-2003	-3.31*(-3.85, -2.48)
2003-2009	-5.57* (-6.24, -5.27)
2009-2014	-4.00* (-4.56, -3.45)
2014-2019	-2.29* (-2.66, -1.69)
**Non-Hispanic White**	
1999-2003	-3.06* (-3.51, -2.35)
2003-2009	-4.67* (-5.25, -4.40)
2009-2014	-2.93* (-3.57, -2.42)
2014-2019	-1.45* (-1.80, -0.84)
**Non-Hispanic Black**	
1999-2003	-2.21* (-2.82, -1.57)
2003-2010	-5.19* (-5.74, -4.93)
2010-2015	-3.08* (-4.20, -2.48)
2015-2019	-1.32* (-1.93, -0.10)
**Non-Hispanic Other**	
1999-2014	-3.93 (-4.41, -3.70)
2014-2019	-1.76 (-2.98, -1.28)
**Hispanic or Latino**	
1999-2005	-3.92* (-4.73,-1.70)
2005-2012	-5.58* (-8.08, -4.17)
2012-2019	-2.28*(-3.14, -0.27)
**Rural Areas**	
1999-2003	-2.41* (-2.93, -1.19)
2003-2007	-4.36* (-5.19, -3.66)
2007-2012	-2.89* (-3.51,-1.19)
2012-2019	-1.17* (-1.61, -0.18)
**Urban Areas**	
1999-2003	-3.23* (-3.70, -2.55)
2003-2009	-5.02* (-5.67, -4.74)
2009-2014	-3.40* (-4.06, -2.74)
2014-2019	-1.72* (-2.10, -0.95)

APC, annual percent change; AAMR, age-adjusted mortality rate.

* Indicates that the annual percentage change is significantly different from zero at α = 0.05.

### Cihd- related mortality stratified by race

**Fig 2:**
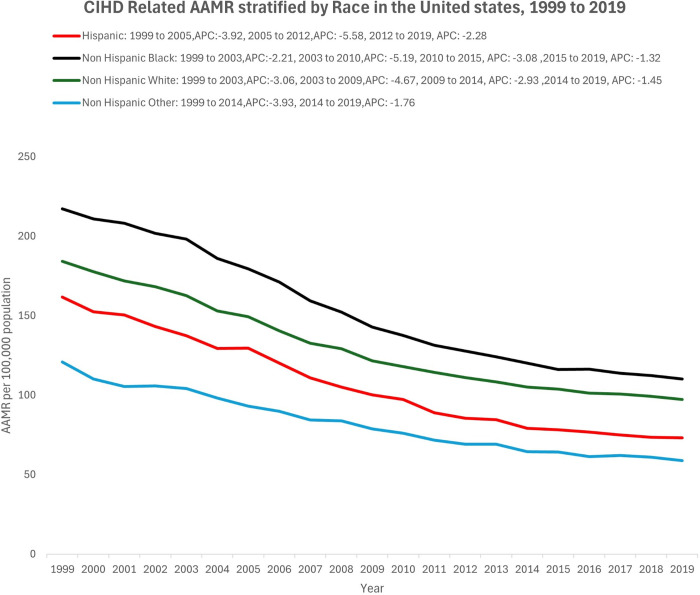
CIHD related age adjusted mortality rate stratified by race in the United States, 1999 to 2019.

When stratified by race, overall AAMR mortality rates were highest in the NHB individuals (148.3, 95% CI: 147.9 - 148.7), followed by NHW individuals (128.5, 95% CI: 128.4 - 128.6), then Hispanics (73.2, 95% CI: 72.1-74.3). The NHO population had the lowest overall AAMR of 58.8(95% CI: 57.5 - 60.0). This trend was consistent over the entire study period (Fig 2, Table 1). For NHB, the AAMR declined consistently over the study period, with an AAPC of -3.39 (95% CI: -3.63 to -3.20, p < 0.01). The decline was segmented into distinct periods: a decrease from 1999 to 2003 (APC: -2.21, p < 0.01), a steeper decline from 2003 to 2010 (APC: -5.19, p < 0.01), a gradual decline from 2010 to 2015 (APC: -3.08, p < 0.05), and a final reduction from 2015 to 2019 (APC: -1.32, p < 0.05). A similar trend was observed in NHW individuals, with an AAPC of -3.12 (95% CI: -3.18 to -3.04, p < 0.01). The steepest decline occurred between 2003 and 2009 (APC: -4.67, p < 0.01). The Hispanic population also showed an overall decline in AAMR (AAPC: -3.94, 95% CI: -4.14 to -3.67, p < 0.01). The study period revealed an initial decline from 1999 to 2005 (APC: -3.92, p < 0.01), followed by a sharp drop from 2005 to 2012, and then a gradual reduction from 2012 to 2019 (APC: -2.28, p < 0.01). For the non-Hispanic Other NHO population, there was an initial sharp decline from 1999 to 2014 (APC: -3.93, p < 0.01), followed by a slower descent from 2014 to 2019 (Fig 2, Table 3).

### Cihd-related mortality stratified by urbanization

**Fig 3:**
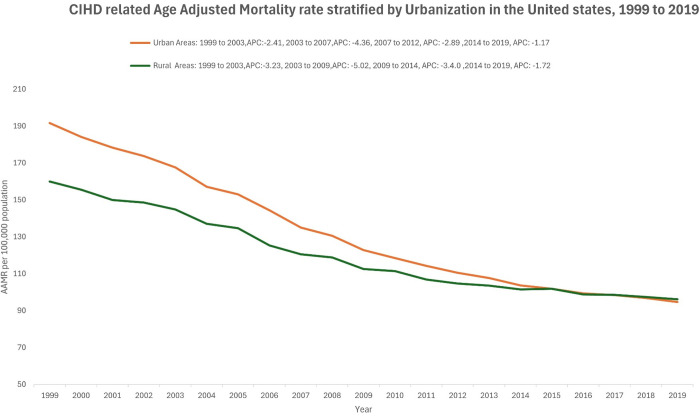
CIHD related age adjusted mortality rate stratified by urbanization in the United States, 1999 to 2019.

Both rural and urban areas experienced a decline in the AAMR over the two-decade study period. At the start of the study, the AAMR in urban areas (191.7, 95% CI: 191.0–192.4) was significantly higher than in rural areas (160.0, 95% CI: 158.6–161.3). However, by the end of the study period, the AAMR for the two regions had converged, with urban areas reporting an AAMR of 94.7 (95% CI: 94.3–95.1) and rural areas reporting an AAMR of 96.1 (95% CI: 95.1–97.0). (Fig 3, Table 1) Urban areas experienced a gradual decline in AAMR throughout the study period, with an AAPC of -3.44 (95% CI: -3.51, -3.36). The steepest decline occurred between 2003 and 2009 (APC: -5.02, p < 0.01). A similar trend was observed in rural areas, with the steepest decline occurring between 2003 and 2007 (APC: -4.36, p < 0.01) and an overall AAPC of -2.49 (95% CI: -2.59 to -2.37, p < 0.01) (Fig 3, Table 3).

## Discussion

Our study provided key insights into a 2-decade mortality trend of CIHD in adults 25 years and above in the United States. The study’s findings highlighted that CIHD contributed to 5,729,619 deaths from 1999 to 2019 with an overall AAMR of 185.6 per 100,000 deaths. The AAMR for CIHD saw a decline across all demographics and urbanization levels studied over the study period, with an AAPC of -2.88. Our study also revealed distinct and persistent disparities when the population was stratified by sex, race/ethnicity, and urbanization levels.

The overall decline in CIHD mortality over the past 20 years can be attributed to a significant reduction in major risk factors through prevention and early diagnosis, and advancement in evidence-based therapies [15]. Widespread public health campaigns and policy making have improved awareness of risk factors, such as smoking, hypertension, hyperlipidemia, and diabetes, leading to better lifestyle modifications and increased adherence to preventive measures [15,16]. In the U.S., the prevalence of smoking has declined over the last 2 decades, which may have contributed to the reduced mortality trends [17,18]. Hypertension, a significant risk factor for CIHD, has also shown a global decline, albeit disproportionately decreasing in high-income countries while increasing in low- and middle-income countries. This decline is attributed to public health interventions, as well as regulatory and behavioral measures such as reduced salt intake and increased consumption of fruits and vegetables [19]. It is however worth stating that in the U.S., the prevalence of significant CIHD comorbidities like diabetes and hypertension has increased over the period [20,21]. The adoption of evidence-based diagnostic tools, such as Quantitative Flow Ratio to assess coronary artery stenosis, advanced imaging techniques like intravascular ultrasound and optical coherence tomography for evaluating coronary plaques, and biomarkers for detecting early-stage atherosclerosis, has also contributed to reduced mortality [5]. Medical therapies, including statins, antiplatelets, and antihypertensive drugs, have significantly reduced the progression of atherosclerosis and CIHD-related mortality [22]. Additionally, advances in interventional cardiology, such as percutaneous coronary interventions (PCI) and coronary artery bypass grafting (CABG), have further improved survival rates [5,23].

Males have a significantly higher AAMR (167.2 vs. 96 per 100,000) and a slower decline in AAPC (-3.91 vs -2.88) for CIHD compared to females. This disparity may be attributed to a combination of biological, behavioral, and systemic factors. Biologically, males exhibit a higher prevalence of calcified plaques and inflammatory markers, such as C-reactive protein, serum fibrinogen, and IL-6, which contribute to an increased risk of major adverse cardiac events [24,25]. In contrast, premenopausal females benefit from estrogen’s protective effects, which enhance endothelial function through its antioxidant effect. Estrogen also lowers LDL levels, reduces HDL, and decreases levels of lipoprotein A, homocysteine, and fibrinogen, thereby reducing the pathogenesis of plaque formation and reducing the progression of CIHD [26,27]. Behaviorally, males demonstrate higher-risk patterns, such as smoking and alcohol consumption, compared to females. Males have over five times the prevalence of smoking and approximately twice the prevalence of alcohol use compared to females [28,29]. In the National Health Interview Survey conducted in the U.S., males were more likely to be current smokers compared to females (17.5% vs 13.6%). Additionally, males have higher rates of established and untreated risk factors for CIHD, such as hypertension and hyperlipidemia. It is estimated that more men than women (51% vs. 41%) have hypertension, and among those, only 18% of men have achieved control compared to 23% of women [19,30]. Males are less likely than women to establish care with primary care providers. Even among men who do establish care, they tend to have fewer contacts with their providers compared to women. This contributes, in part, to delayed diagnosis and treatment of underlying risk factors for CIHD [31]. These factors highlight the critical need for targeted preventive measures to reduce CIHD-related mortality in men.

Our study revealed that NHB had the highest CIHD-related AAMR compared to all the other racial groups. This trend was observed over the entire study period, and it is consistent with previously published IHD and cardiovascular disease trends [7,32]. NHBs over the decades have been disproportionately affected by socioeconomic inequities such as reduced access to healthcare, low insurance coverage rates, and reduced access to healthcare [33]. To further compound this risk, NHBs have a high prevalence of modifiable cardiovascular risk factors like obesity, sleep deprivation, diabetes, and hypertension, contributing to an increased mortality rate in CIHD [34–36]. Additionally, living in disadvantaged neighborhoods is an independent risk factor for coronary heart disease, and black individuals are more than four times as likely as White individuals to live in these areas, further increasing their risk and subsequent mortality [37,38]. Hispanics had a lower CIHD-mortality compared to NHB and NHW. This trend aligns with the Hispanic paradox, which suggests that despite having lower socioeconomic status and a higher prevalence of cardiovascular disease, Hispanics exhibit lower cardiovascular-related mortality [39].

Over the past two decades, CIHD-related mortality rates in urban and rural areas have converged. Initially, AAMRs were higher in urban areas; however, by the end of the study period, these differences had largely diminished. Historically, several countries, including the U.S., have reported elevated IHD mortality in urban areas compared to rural areas. This was attributed to urban-specific risk factors such as higher population densities, increased exposure to environmental pollutants, and unhealthy lifestyle behaviors, including sedentary habits and increased consumption of highly processed diets [40–42]. However, recent studies, including our findings, suggest a reversal of this trend, with rising CIHD mortality in rural areas and a decline in urban regions [42,43]. Urban areas have witnessed improved technological advancement, significant improvements in healthcare infrastructure, greater access to specialized medical services, and the implementation of effective public health interventions targeting underlying cardiovascular risk factors. These advancements have facilitated a more pronounced decline in CIHD mortality in urban populations [42,44]. Conversely, rural areas have faced persistent healthcare challenges over the 2-decade period. Rural areas have limited access to medical care and a shortage of healthcare professionals, with reported increased rural hospital closures [43,45,46]. Additionally, rural areas have a higher burden of modifiable CIHD risk factors like increasing physical inactivity, obesity, smoking, diabetes, and hypertension [47–50]. Our findings, therefore, highlight the need for targeted public health interventions and resource allocation to address rural populations’ unique challenges in managing and preventing CIHD.

Socioeconomic factors, while not directly assessed in this study, remain pivotal in influencing IHD mortality. Studies have demonstrated that individuals with lower educational attainment are at a substantially elevated risk of cardiovascular mortality, including IHD, primarily driven by behavioral risk factors such as smoking, physical inactivity, and obesity [51,52]. Furthermore, lower education levels often intersect with other social determinants, including reduced health literacy, limited access to healthcare, and increased socioeconomic adversity, collectively compounding cardiovascular risk [53,54]. Occupational factors also independently elevate the risk of IHD, with variables such as psychosocial stress, extended working hours (≥ 55 hours/week), night shifts, and exposure to physical and chemical hazards being significant contributors [55–57]. Thus, addressing these socioeconomic and occupational disparities is essential for mitigating IHD mortality and promoting equitable cardiovascular health outcomes.

### Limitations

Our study has certain limitations. First, the study analyzed mortality trends based on ICD-10 codes from death certificates in the CDC WONDER database, so there may be a risk of misclassification bias or underreporting of CIHD. Second, the CDC WONDER database lacks baseline comorbidities, cardiovascular risk factors, and laboratory values, which limits our ability to explore potential disparities in mortality trends. Third, the absence of socioeconomic indicators such as income, educational level, occupation, and access to healthcare in the database restricts us from identifying potential factors that may have influenced the trends. Lastly, while the CDC WONDER database allows for national trend analysis, individual-level analyses and causal inferences are not possible due to its aggregated nature.

## Conclusion

Over the last 2 decades, CIHD-related mortality for individuals 25 years and above, in the U.S., has declined overall and for all demographic groups. However, significant disparities persist, particularly in the male gender and the NHB population, with similar AAMR between rural and urban areas at the end of the study period. It is therefore crucial to understand the factors contributing to this trend and focus. These findings highlight the need for a deeper understanding of the factors driving these disparities and targeted public health interventions to improve CIHD-related mortality outcomes in these vulnerable populations.
